# Extraordinary Success in Ovariotomy

**Published:** 1876-08

**Authors:** 


Prof. Koberle—Extraordinary Success in Ovariotomy.—A correspondent of the *Allgem, Wien. Med. Zeit.*, No. 20, 1876, who had been visiting K. at Strasburg, mentions many interesting facts as to his practice. His first ovariotomy was done in 1862. He had never seen it performed, and may be called antididactos. He has now performed 250. Of 40 cases operated on in two years, he only lost 2, the 38 having all recovered perfectly. He operates without any assistance except a sister of mercy.(?) He uses no water, sponges, or disinfectants. The wound is cleansed only with balls of charpie. He usually visits the patient twice daily after the operation, and does the dressing with his own hands. Since Strasburg has fallen into the hands of the Germans, K. has resigned his professorship, receiving no pension from France, and declining employment from Germany, whose authority he obeys from compulsion. According to this correspondent, he is a man of great general accomplishment, being a practical sculptor, turner, mechanic, gardener, and indeed master of many trades. —*The Doctor*, June 1, 1876.

